# 3D-QSAR Studies, Molecular Docking, Molecular Dynamic Simulation, and ADMET Proprieties of Novel Pteridinone Derivatives as PLK1 Inhibitors for the Treatment of Prostate Cancer

**DOI:** 10.3390/life13010127

**Published:** 2023-01-02

**Authors:** Mohammed Er-rajy, Mohamed El fadili, Hamada Imtara, Aamir Saeed, Abid Ur Rehman, Sara Zarougui, Shaef A. Abdullah, Ahmad Alahdab, Mohammad Khalid Parvez, Menana Elhallaoui

**Affiliations:** 1 LIMAS Laboratory, Faculty of Sciences Dhar El Mahraz, Sidi Mohamed Ben Abdellah University, Fez 30050, Morocco; 2 Faculty of Arts and Sciences, Arab American University Palestine, Jenin B.P. Box 240, Palestine; 3 Department of Bioinformatics, Hazara University Mansehra, Dhodial 21120, Pakistan; 4 Department of Biotechnology and Genetic Engineering, Hazara University Mansehra, Dhodial 21120, Pakistan; 5 Department of Cardiology, Karlsburg Hospital, 17495 Greifswald, Germany; 6 Institute of Pharmacy, Clinical Pharmacy, University of Greifswald, Friedrich-Ludwig-Jahn-Street 17, 17489 Greifswald, Germany; 7 Department of Pharmacognosy, College of Pharmacy, King Saud University, Riyadh 11451, Saudi Arabia

**Keywords:** 3D-QSAR, PLK1 inhibitors, molecular docking, dynamic simulation, anti-cancer

## Abstract

Overexpression of polo-like kinase 1 (PLK1) has been found in many different types of cancers. With its essential role in cell proliferation, PLK1 has been determined to be a broad-spectrum anti-cancer target. In this study, 3D-QSAR, molecular docking, and molecular dynamics (MD) simulations were applied on a series of novel pteridinone derivatives as PLK1 inhibitors to discover anti-cancer drug candidates. In this work, three models—CoMFA (Q² = 0.67, R² = 0.992), CoMSIA/SHE (Q² = 0.69, R² = 0.974), and CoMSIA/SEAH (Q² = 0.66, R² = 0.975)—of pteridinone derivatives were established. The three models that were established gave $R_{\mathrm{pred}}^{2}$ = 0.683, $R_{\mathrm{pred}\;}^{2}$= 0.758, and $R_{\mathrm{pred}\;}^{2}$= 0.767, respectively. Thus, the predictive abilities of the three proposed models were successfully evaluated. The relations between the different champs and activities were well-demonstrated by the contour chart of the CoMFA and CoMSIA/SEAH models. The results of molecular docking indicated that residues R136, R57, Y133, L69, L82, and Y139 were the active sites of the PLK1 protein (PDB code: 2RKU), in which the more active ligands can inhibit the enzyme of PLK1. The results of the molecular dynamic MD simulation diagram were obtained to reinforce the previous molecular docking results, which showed that both inhibitors remained stable in the active sites of the PLK1 protein (PDB code: 2RKU) for 50 ns. Finally, a check of the ADME-Tox properties of the two most active molecules showed that molecular N° 28 could represent a good drug candidate for the therapy of prostate cancer diseases.

## 1. Introduction

Among the most infectious and dangerous diseases in recent years is cancer disease, which is manifested by the abnormal regulation of different pathways; it remains the second most prevalent disease and one of the major health problems in the world [1]. The research into anti-cancer drugs targeting key factors is essential to regulate this major problem [2]. Among the methods used to discover drug candidates are computational methods to reduce the cost of drugs and increase their effectiveness [3]. Polo-like kinases (PLKs), a serine–threonine kinase, have five family members (PLK1-5) that play a key role in mitosis and have been proven to be necessary for centrosome maturation and bipolar spindle formation [4]. PLK1 is the most investigated of all PLK family members. PLK1 overexpression has been found in many types of different cancers (lung cancer, prostate cancer, colon cancer, etc.) and it plays an essential role in cell proliferation. However, PLK1 has been determined to be a broad-spectrum anti-cancer target [5].

A series of novel pteridinone derivatives were synthesized and evaluated in their biological activity by Zhiwei Li et al. [6]. To identify the best PLK1 enzyme candidate inhibitors for the treatment of prostate cancer, this series is undergoing a molecular modeling study. Quantitative structure–activity relationship (QSAR), molecular docking, molecular dynamic (MD), and chemical absorption, distribution, metabolism, excretion, and toxicity (ADMET) molecular property studies have been very important modeling methods to generate predictive and robust models to predict and study new drug development candidates with a reasonably low economic impact [7].

The comparative molecular field analysis (CoMFA) and comparative molecular similarity indices analysis (CoMSIA) methodologies permit a correlation between the dependent variations (pIC_50_) and the different molecular properties in order to establish a good mathematical model [8]. To check the predictive capacity and robustness of the different proposed models, such as CoMFA and CoMSIA, external and internal validations were discussed [9].

The molecular docking approach, used to model the interaction between a small molecule and a protein at the atomic level, allowed us to characterize the behavior of small molecules in the binding site of target proteins and to resolve fundamental biochemical processes [10]. Once the docking results were obtained, it was necessary to determine the structural behavior of the most active molecular characteristics, such as structural orientation, any biological influence in the structure, the parameters that can force the achievement of the biological activity of the molecule, and others [11]. Then, we performed molecular dynamics simulations to analyze and deepen the details of the interaction and stability of the two docked ligands in the target proteins [12].

**Figure 1:**
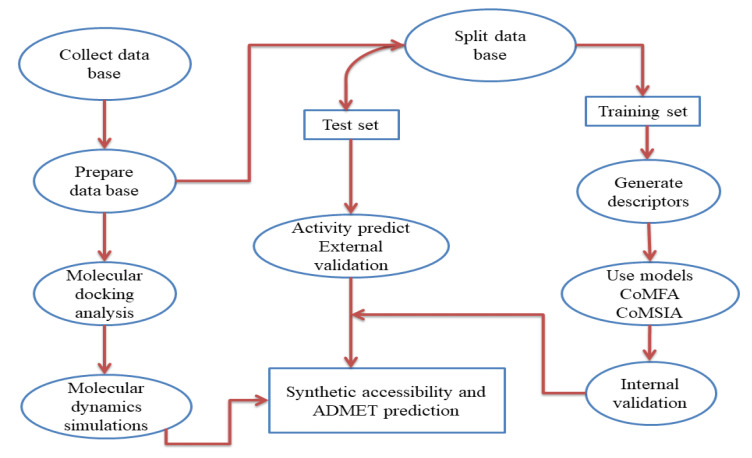
Flowchart of the different studies used.

Finally, we evaluated the ability of both compounds to successfully act as drug candidates, tested by pharmacokinetic and pharmacodynamic parameters (ADMET and Lipinski rule) [13]. Figure 1 presents a flow chart of the QSAR model development method and all the different steps used in the present work.

## 2. Materials and Methods

### 2.1. Database and Biological Activity

To build the different CoMFA and CoMSIA models, we based them on a set of experimental data (28 derivatives, Table 1) synthesized by Zhiwei Li et al. [6]. To evaluate their anti-cancer biological activity (IC_50_), we divided the experimental data set (80% training set (22 derivatives) and 20% test set (6 derivatives)) [7] and the training set to construct a model and used the test set to evaluate the performance of the built model.

### 2.2. Molecular Alignment and Generation of the Models

First of all, it was necessary to align all the molecules because molecular alignment is one of the most important steps for the generation of the CoMFA and CoMSIA models [14]. In the database where we grouped all the molecules of the new pteridinone derivatives, we performed a rigid distill alignment using SYBYL-X 2.1 software [15]. The database of all molecules was minimized by using the standardized Tripos force field and employing the Gasteiger–Huckel atomic partial charges. Furthermore, 0.005 kcal/mol Å was set as the standard convergence parameter for the Powell gradient algorithm, and 1000 rounds of iterations were performed to produce a more stable configuration of the molecule [16]. Secondly, field studies of the CoMFA and CoMSIA descriptors were computed for each lattice, with a grid distance of 1 Å and extending to 4 Å points, in all three coordinates within the defined region [17]. Steric (S), electrostatic (E), acceptor hydrogen bonding (A), and hydrophobic (H) fields were calculated using the standard Tripos force field and a Van Der Waals potential, as well as the Coulombic terms [18]. A sp^3^ hybridized carbon (C) atom with a charge of +1e was utilized as the probe atom, and steric and electrostatic field energy values were truncated at 30 kcal/mol [19]. The attenuation factor and filter factor values for the column were 0.3 and 2 kcal/mol for the steric and electrostatic fields [20].

Finally, the partial least-squares (PLS) method allowed for the correlation between the different CoMFA and CoMSIA fields, which included the value of the biological activity of pteridinone derivatives [21]. This method is usually used in 3D-QSAR studies to determine the various statistical values, such as the optimal number of components (NOC), the cross-validated coefficients (Q²), the conventional coefficient (R²), F-statistic values (F), and the standard error of estimation (SEE). An accurate model means higher R² and Q² statistical parameters and the smallest possible SEE [22]. To estimate the predictive capabilities of the 3D-QSAR models, the biological activity of the external test set (5 molecules) was predicted using the resulting PLS models, and the predictive correlation coefficient ($R_{\mathrm{pred}}^{2}$), based on the molecules in the test set, must be greater than 0.6 [23]. The QSAR model is considered good when the Q² value is greater than 0.5 [24]. During this analysis, SAMPLES remained disabled, and column filtering was set to 2.0 kcal mol^−1^ to speed up the analysis [25]. The leave-one-out (LOO) method involved removing one of the individual compounds from the training set and then predicting the activity of each removed compound to verify that the results of the CoMFA and CoMSIA models were predictive for the compounds that were not in the training set [26].

### 2.3. Molecular Docking

The objective of molecular docking is to give a prediction of the structure of the ligand-receptor complex using computational methods. Docking can be performed in two interdependent steps: first, sampling the conformations of the ligand in the active site of the protein; then, classifying these conformations via a scoring function. The software we used to perform the molecular docking, such as that discovery in 2021 to delete molecules of water and view the ligand/protein interaction [27], was Auto Dock Tools 1.5.6 [28], and we used Vina to execute molecular docking [29]. We used the co-crystalline structure BI-2536/PLK1 (PDB code: 2RKU, with a resolution of 1.95 Å) [13] as a docking model and performed molecular simulation docking of the most active molecules with PLK1 [30,31]. Key amino acid residues that facilitated docked ligand binding to the PLK1 active site were R136, R57, Y133, L69, L80, H138, Y67, Y82, and E132. After the preparation of the ligand and the receptor, we effected molecular docking on the two most active molecules, N° 17 and 28.

### 2.4. Molecular Dynamic (MD)

Based on the molecular docking results, the two best-docked ligands with the highest activity were chosen for molecular dynamics (MD) simulations to identify the molecular recognition between the ligand and the protein [32]. MDs were performed for 50 nanoseconds using GROMACS 5.0 software and the GROMOS9643a1 force field [33]. The SOC water model was chosen to simulate the MD in explicit solvation [34]. The ligand loading parameters were obtained from the Dundee prodrg 2.5 servers [35]. Other input parameters were selected, such as the SOC water model, the type of triclinic box, and the type of salt (Na^+^, Cl^−^), to be neutralized [36]. The system was equilibrated at a temperature of 300 K and a pressure of 1 bar, with canonical NVT and isobaric NPT sets, respectively [37]. The MD simulations were performed for a time of 50 ns, with the temperature and pressure stable, a time step of 2 fs, and a long-range interaction threshold of 1 nm [38].

### 2.5. Synthetic Accessibility and ADMET Prediction

After the stability study of the two most active molecules (molecule N° 17 and 28), it was necessary to study their pharmacokinetic and pharmacodynamics properties. First, it was necessary to verify the synthetic accessibility of these compounds, and second, it was necessary to study the pharmaceutical properties of each compound. The pkCSM [39] and SwissADME servers [40] were used to evaluate the synthetic accessibility and pharmaceutical properties of the proposed compounds [41].

## 3. Results and Discussion

### 3.1. Distill Rigid Alignment

**Figure 2:**
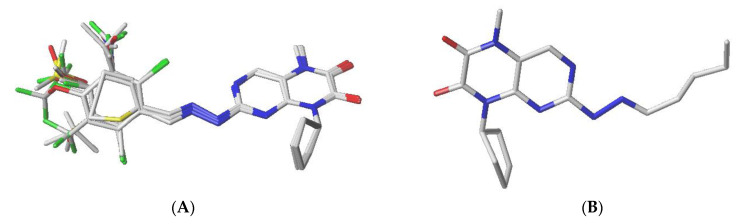
Superposition of database molecules (**A**), on the core molecule (**B**).

The structural alignment of molecules is critical to both the predictive accuracy of the 3D-QSAR model and the reliability of the contour models. The database of pteridinone-derived molecules was aligned on the common core using the distill rigid alignment technique in Sybyl X-2.1. Molecular N° 28 (most active) was considered a template. Based on Figure 2, all 3D molecular structures were superimposed (Figure 2A) on the common core (Figure 2B).

### 3.2. Generation of the CoMFA and CoMSIA Models

The Table 2 shows the predicted and observed activity values of the different CoMFA and CoMSIA models and their residuals.

The 3D-QSAR models were suggested to quantitatively explicate and predict the different effects of the molecules on the anti-proliferative activities of a series of twenty-eight pteridinones. The variables in the training set were put into PLS cross-validation analysis to identify the proper statistical metrics for every module. The Table 3 shows the results for the different statistical modules and their own parameters. The following table demonstrates the results for the different statistical modules and their own parameters.

The percentage of CoMFA model contributions of the two fields (steric and electrostatic) explained 81.4% and 18.6% of the variance, respectively, the values of $Q_{\mathrm{cv}}^{2}$ and R² were 0.67 and 0.992, respectively, the optimal number of principal components (NOC) used was 9, the F-value was 27.47, and the lower value of SEE was equal to 0.035.

In the CoMSIA study, the evaluation analysis of the three selected models had various combinations of the fields, such as stereoscopic (S), hydrophobic (H), electrostatic (E), and hydrogen bond acceptor (A). According to Table 3, among the different field combinations chosen, the best models were CoMSIA/SEH and CoMSIA/SEAH, which obtained the highest $Q_{\mathrm{cv}}^{2}$ values of 0.69 and 0.66, respectively, with principal components 7 and 7, respectively, F-values of 15.52 and 12.30, respectively, $R_{\mathrm{cv}}^{2}$ values equal to 0.974 and 0.975, respectively, and lower values of SEE equal to 0.059 and 0.057, respectively.

The results showed that steric, electrostatic, hydrogen bond acceptor, and hydrophobic fields played an important role in these models. Among these four fields, the steric and hydrophobic fields were the most important interactions between the ligand and the receptor protein.

### 3.3. External Validation

From the results in Table 4, it can be seen that the four models, CoMFA, CoMSIA/SEH, and CoMSIA/SEAH, had better external prediction coefficients than the three models ($R_{\mathrm{pred}}^{2}$), which were operated to validate the external predictive abilities of the three models. The $R_{\mathrm{pred}}^{2}$ values of the three models were 0.683, 0.758, and 0.767, respectively. Therefore, the three models had Q² values greater than 0.5 [23]. Regressions of the predicted pIC_50_ versus the observed pIC_50_ or the predicted pIC_50_ versus the observed pIC_50_ through the origin should be characterized by the fact that K or K’ (slopes of corresponding regression lines) is close to 1 [42]. According to Table 4, the two models chosen for external validation were CoMSIA/SEH and CoMSIA/SEAH, which obtained values of K and K’ of 0.923 and 1.012, respectively, so they were close to 1. Therefore, these two models (3D-QSAR) that we chose are acceptable. Then, we used them to predict the activity of a test molecule.

### 3.4. Analyzation of the CoMFA and CoMSIA Contour Charts

**Figure 3:**
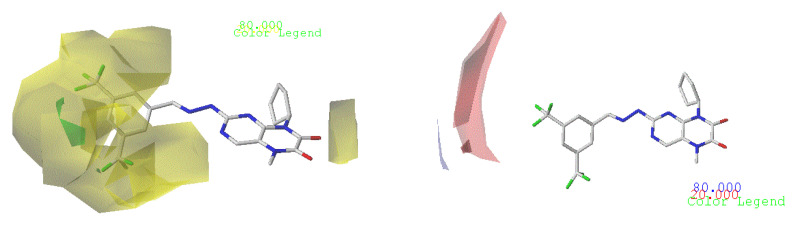
Stereoscopic (S) and electrostatic (E) contour charts.

**Figure 4:**
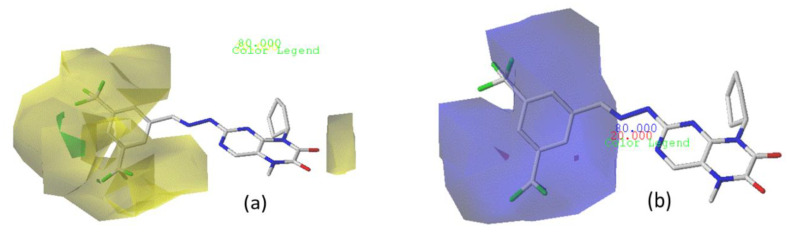

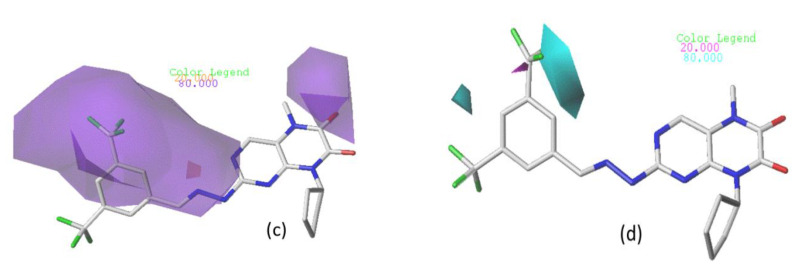
Stereoscopic (**a**), electrostatic (**b**), hydrogen bond acceptor (**c**), and hydrophobic (**d**) contour diagrams.

To show the details contained in the two 3D-QSAR models, we selected the more active molecule in the series (molecule N° 17) to visualize the different fields on the molecule. The different fields of the CoMSIA and CoMFA contour charts are presented in Figure 3 and Figure 4.

#### 3.4.1. CoMFA Contour Chart

In the CoMFA model of the two fields, stereoscopic and electrostatic are presented in Figure 3.

The two figures represent the two electrostatic and stereoscopic contour plots from the CoMFA study that can help provide information about the regions that can decrease or increase the biological activity of pteridinone derivatives. At the steric field level, only a few green outline parts are located near the meta-position of the benzene ring, which means that the para-position increases the inhibitory activity of the more active compound. On the other hand, a large yellow contour portion that decreases the inhibitory activity of the more active compound originates from the pteridinone molecule substituents. In the electrostatic field, the blue contours indicate that it is positively charged and favors the inhibitory activity of this ligand, while the red contours indicate that this region is negatively charged and disadvantages the inhibitory activity of this ligand. After studying the figure, we found that both fields were farther from the ligand because of the ligand that has no charge.

#### 3.4.2. CoMSIA/SEA Contour Chart

In the CoMSIA/SEA model, four different fields, stereoscopic, electrostatic, hydrogen bond acceptor, and hydrophobic fields, were checked. The four contour charts are presented in Figure 4.

The CoMSIA steric contour chart (Figure 4a) of the most active molecule presented similar results to the CoMFA contour chart. However, at the electrostatic contour chart levels (Figure 4b), we observed a large blue contour covering the R substitutions, indicating that the selection of large substitution groups (CF3) is necessary for this region to increase the inhibitory activity. We also observed a small red contour. At the acceptor hydrogen bonding contour chart level (Figure 4c), we observed a large purple contour covering the meta-position of R substitutions, indicating that the selection of bulky substitution groups at the meta-position is necessary for this region to increase inhibitory activity. We also observed a small orange contour away from the R substituent. At the hydrophobic binding contour map level (Figure 4d), we observed a small cyan contour covering the meta-position of R substitutions, indicating that the selection of bulky substitution groups at the meta-position is necessary for this region to increase inhibitory activity. We observed almost no magenta contour around the R substituent.

Therefore, it can be concluded that the meta-position of substituent R allows for an increase in the inhibitory activity of the chosen molecule if this position has an attractive and bulky group.

### 3.5. Molecular Docking

**Figure 5:**
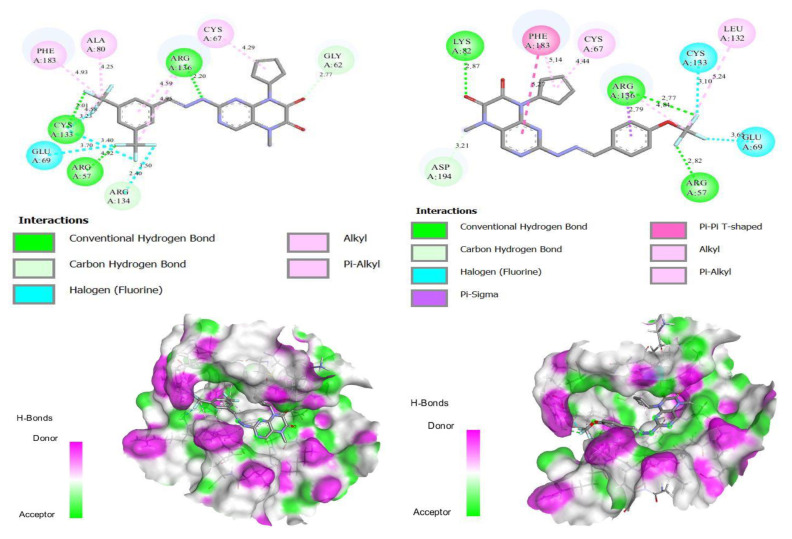
The molecular docking results of the two molecules selected (2 and 3-dimensional visualization).

To explain and understand the interaction between the most active molecule and its protein, we performed a molecular docking of the two most active molecules. The results found by molecular docking for the two selected compounds (molecule N° 17 and 28) are presented in Figure 5.

The first visual surveillance of the two results shows that there are three hydrogen bonds with residues R136, R57, and Y133, with a distance match to 2.20, 4.94, and 2.01, respectively. There is one halogen bond with residue L69, with a distance match to 3.70, and three alkyl bonds, L80, H183, and Y67, with a distance match to 4.25, 4.95, and 4.29, respectively, in molecule N° 17. In molecule N° 28, we also observed three hydrogen bonds with residues R136, R57, and Y82, with an equal distance of 2.79, 2.82, and 2.87, respectively, and two halogen bonds with residues L69 and Y139, with match distances 3.65 and 3.10, respectively. There are two alkyl bonds with residues E132 and Y67 and a single Pi–Pi bond with residue H183. Thus, these different interactions between the molecules and their protein mean that the two molecules have a greater inhibitory effect. The overall interactions are similar to the interactions observed between a co-crystallized ligand and protein PLK1.The results suggest that the docking result is reasonable and can be used for further simulations and analysis, such as the MD simulation.

### 3.6. Molecular Dynamics Simulation

**Figure 6:**
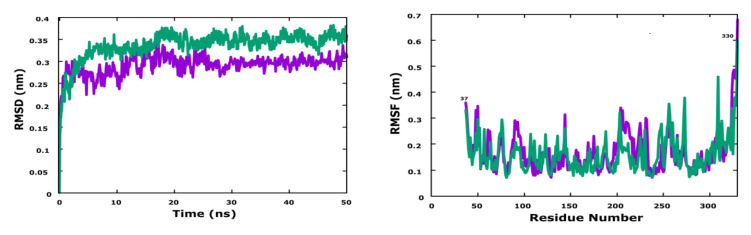
RMSD and RMSF of molecular dynamics results.

The two most active ligands (molecules N° 17 and 28) were chosen for dynamic molecular simulation for 50 ns to examine their stability, with respect to enzyme PLK1. The conformational changes of the two ligands are shown in the Figure 6.

To study the dynamic of the protein–ligand interactions of two docked molecules, a 50 ns MD simulation was run using two complexes (PLK1-molecule N° 17 (violet) and PLK1-molecule N° 28 (green)) designed for the protein to ensure the predicted binding stability of the complex system. From Figure 6, it can be seen that the RMSD of the complex PLK1-molecule N° 17 fluctuated between 0.22 and 0.30 nm during the MD simulations, and the average RMSD was found to be 0.28 nm, from 0 to 30 ns. The curve of the complex increased slightly to the value of about 0.28 nm, and then an equilibrated system was obtained in the complex for the remaining time.

The RMSD analysis indicated that for the complex PLK1-molecule N° 28, the protein fluctuated between 0.22 and 0.36 nm during the MD simulations, and the average RMSD was found to be 0.3 nm. The RMSD analysis indicated that the designed molecules N° 28 and N° 17 formed a stable complex with the protein throughout the simulations.

To study the impact of the binding of the two designed molecules on the internal dynamics of the target protein for 50 ns, the RMSF values were also calculated. A maximum fluctuation of 0.68 nm was noticed in the loop region of residue 330 for both molecules.

**Figure 7:**
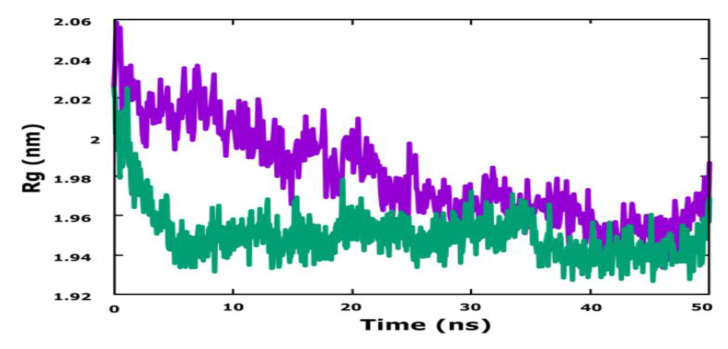
The radius of gyration of the molecular dynamics results.

The radius of gyration (Rg) (Figure 7) represents the change in the compactness of the protein structure over time. For the complex PLK1-molecule N° 28, during the first 8 ns, the values of the Rg varied between 1.94 nm and 2.02 nm. After this time, until the end of the simulation, the values remained reasonably stable in the range of 1.94 to 1.96. The complex PLK1-molecule N° 17 Rg values fluctuated over time between 1.95 nm and 2.06 nm.

Therefore, the Rg plot shows that there is no major change in the folding compactness of the target protein after the binding of molecule N° 28, but there is a small change in the levels of the target protein after the binding of molecule N° 17.

After studying the MD simulation diagram, the results obtained reinforced the previous molecular docking results. Thus, the two chosen ligands formed dynamically stable interactions with their proteins during the simulation time of 50 ns, as there was no great variation in their characteristics.

### 3.7. Synthetic Accessibility and Lipinski Rules

The synthetic accessibility allowed for the evaluation of the ease of synthesis in the best molecules chosen (molecules N° 17 and 28). Then, we evaluated the different properties of the five Lipinski’s rules, which allowed us to determine whether a biologically active chemical was probable to have the chemical and physical characteristics be orally bioavailable. Table 5 lists the different synthetic availability properties and their Lipinski properties.

The results obtained in Table 5 indicate that molecule N° 28 checked all the rules of Lipinski, Veber, and Egan. That means the selected molecule has good oral bioavailability. We also evaluated the synthetic accessibility, and from the result obtained, we concluded that this compound has easy-to-synthesize characteristics. Molecule N° 17 has characteristics that are almost the same as molecule N° 28; it only has a small difference at the molecular mass level that exceeds the norm of 500. In conclusion, we can say that molecule N° 28 has a small attrition rate when tested in clinical trials and has a higher chance of success in reaching the commercial phase.

### 3.8. The Various ADMET Properties

To avoid failing clinical tests due to toxicity or poor pharmacokinetics, after checking the similarity of the drugs, the two most active molecules, N° 17 and 28, were subjected to ADMET prediction (Table 6) to verify that the pharmacokinetic (absorption, distribution, metabolism, and excretion) and pharmacodynamic (efficacy and toxicity of the drug) properties of the molecules passed the study of similarity for drugs.

The water solubility of a compound reflects the solubility of the molecule in water at 25 °C [43]. The solubility in water is given in log (mol/L). If the solubility value is less than zero [44], we can say that the compound is very soluble, which means that both compounds are highly soluble in water. The intestinal absorbance values were very high, above 85%, which means that both compounds had good absorbance. Caco-2 permeability is frequently used to predict the absorption of orally administered medication; a molecule considered highly permeable, Caco-2 should give predicted values at 0.9 [45], so according to the Caco-2, the values of two selected compounds can be classified as highly permeable Caco-2. The volume of distribution (VDss) is used to analyze the distribution of drugs in different tissues in vivo. If the log (VDss) value is less than −0.15, the volume of distribution is considered relatively poor, and when the log (VDss) value is superior to 0.45, the volume of distribution is classified as relatively high [46]. Both compounds had a VDss value less than −0.15, so both compounds had a low volume of distribution for drug distribution in various tissues in vivo. In terms of the central nervous system (CNS) permeability index, molecules with values (LogPS) greater than −2 are considered to be capable of penetrating the CNS, and values of LogPS less than −3 are incapable of penetrating the CNS [47]. From the table, we can summarize that all compounds of N° 17 are capable of penetrating the CNS (values less than −3), but compound N° 28 is not capable of penetrating the CNS. CYP2D6, CYP3A4, CYP1A2, CYP2C19, and CYP2C9 are isoforms of cytochrome P450, which is a crucial detoxification enzyme in the human body and is responsible for altering drug pharmacokinetics [48]. Cytochrome P450 isoenzymes play an important role in drug metabolism in the liver [49]. From the results obtained in Table 6, the two tested molecules did not become substrates of CYP2D6 or substrates of CYP3A4. At the same level, most of the compounds tested were not inhibitors of CYP1A2, CY2C9, and CYP2C19. Excretion refers to the process by which the body gets rid of waste/toxic products. The drug excretion process can be achieved by either the kidney and/or the liver, where drugs are eliminated in the forms of urine or bile, respectively [50]. The total clearance of the drug gives a general view on the half-life of the drug; the lower its value, the higher the half-life of the compound [51]. The two compounds had a low clearance value, which means that the half-life of these two compounds is high. Predicting the toxicity of compounds is an important part of the drug design development process. Computational estimates of toxicity are not only faster than determining toxic doses in animals, it can also help reduce the number of animal experiments [52]. To check the toxicity of two molecules, the Ames toxicity test was performed, and skin sensitization was checked. From Table 6, it can be concluded that both compounds were not toxic. Thus, based on the drug similarity studies and ADMET, we chose molecule N° 28 as the PLK1 enzyme inhibitor because it checks almost all of the similarity properties of a drug. However, it is necessary to make further studies in the area where this compound is a drug.

## 4. Conclusions

This study focuses on a series of twenty-eight novel pteridinone derivatives as PLK1 inhibitors. In the first step, we constructed three models and examined them using external and internal validation to identify the radical of the molecule drifts that had an influence on the biological activity of a molecule.

The models CoMFA (Q² = 0.67, R² = 0.992), CoMSIA/SHE (Q² = 0.69, R² = 0.974) and CoMSIA/SEAH (Q² = 0.66, R² = 0.975) models were used to study molecular modeling. The three models were satisfactory according to the results of the statistical validation ($R_{\mathrm{pred}}^{2}$ value of CoMFA, CoMSIA/SHE and CoMSIA/SEAH models is 0.683, 0.758, and 0.767 respectively). We used these models to predict the activity of the molecules in the test set, and then we can use these models to predict the activity of new molecules as PLK1 inhibitors for prostate cancer treatment.

A molecular docking study was performed to identify the type of binding between the most active ligand and the PLK1 inhibitor. The key amino acids affecting the activity of these inhibitors, such as R136, R57, and Y133, easily formed hydrogen bonds with the selected small molecules and a halogen bond with the L69 residue, so these different bonds could allow the PLK1 inhibitors to maintain stability in the binding site.

Thus, the two selected ligands formed dynamically stable interactions with their protein during the 50 ns simulation time. Finally, an ADMET prediction of two ligands showed that only compound N° 28 could become a good drug candidate for cancer drug development.

## Figures and Tables

**Table 1:** Structures and IC_50_ values of novel pteridinone derivatives.

Comp	R	IC_50_	pIC_50_	Comp	R	IC_50_	pIC_50_ *
1	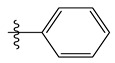	48.20	4.316	15	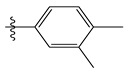	39.03	4.818
2 *	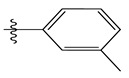	53.59	4.270	16	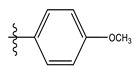	26.25	4.408
3	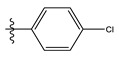	8.42	5.074	17	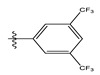	8.20	4.580
4	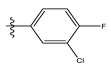	8.55	5.068	18	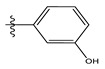	36.30	5.086
5 *	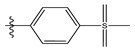	26.59	4.575	19 *	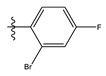	9.25	4.440
6	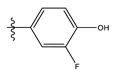	85.15	4.069	20	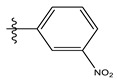	20.32	5.033
7	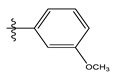	20.68	4.684	21 *	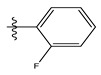	27.59	4.692
8	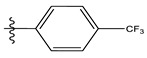	23.61	4.626	22 *	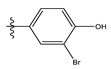	11.58	4.559
9	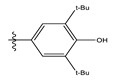	17.72	4.751	23	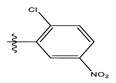	9.26	4.936
10	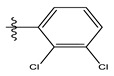	17.20	4.764	24	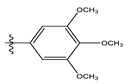	17.50	5.033
11	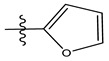	72.16	4.141	25 *	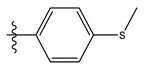	21.03	4.756
12	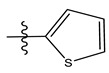	75.63	4.121	26	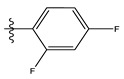	13.17	4.677
13	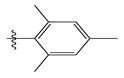	13.88	4.857	27	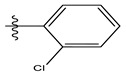	16.31	4.880
14	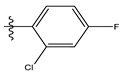	15.18	4.818	28	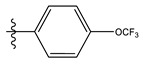	7.18	4.787

* Test set, pIC_50_ = 6-log_10_ (IC_50_).

**Table 2:** The observed/predicted activity and their residuals of different models.

	pIC_50_ obs *	pIC_50_ Predict					
N°		CoMFA	Residual	CoMSIA/SEAH	Residual	CoMSIA/SEH	Residual
1 *	4.316	4.329	−0.013	4.263	0.053	4.259	0.057
2	4.271	4.276	−0.005	4.327	−0.056	4.33	−0.059
3 *	5.074	4.471	0.603	4.492	0.582	4.486	0.588
4 *	5.068	4.606	0.462	4.678	0.39	4.678	0.39
5	4.575	4.545	0.03	4.599	−0.024	4.606	−0.031
6	4.07	4.116	−0.046	4.155	−0.085	4.158	−0.088
7 *	4.684	4.328	0.356	4.315	0.369	4.308	0.376
8	4.626	4.631	−0.005	4.605	0.021	4.603	0.023
9	4.751	4.745	0.006	4.755	−0.004	4.745	0.006
10	4.764	4.779	−0.015	4.129	0.635	4.784	−0.02
11	4.141	4.123	0.018	4.122	0.019	4.124	0.017
12	4.121	4.152	−0.031	4.178	−0.057	4.176	−0.055
13	4.857	4.883	−0.026	4.835	0.022	4.833	0.024
14	4.818	4.831	−0.013	4.835	−0.017	4.849	−0.031
15	4.409	4.343	0.066	4.44	−0.031	4.439	−0.03
16	4.581	4.58	0.001	4.556	0.025	4.559	0.022
17	5.086	5.091	−0.005	5.126	−0.04	5.12	−0.034
18	4.440	4.394	0.046	4.286	0.154	4.282	0.158
19	5.034	5.028	0.006	4.986	0.048	4.986	0.048
20	4.692	4.717	−0.025	4.692	0.000	4.69	0.002
21	4.559	4.549	0.01	4.509	0.050	4.511	0.048
22 *	4.936	4.534	0.402	4.529	0.407	4.52	0.416
23	5.033	4.996	0.037	5.054	−0.021	5.058	−0.025
24	4.757	4.795	−0.038	4.749	0.008	4.755	0.002
25	4.677	4.717	−0.04	4.68	−0.003	4.684	−0.007
26	4.880	4.864	0.016	4.868	0.012	4.866	0.014
27	4.787	4.797	−0.01	4.755	0.032	4.752	0.035
28	5.144	5.126	0.018	5.172	−0.028	5.168	−0.024

*: Test; obs: observed.

**Table 3:** The results of PLS cross-validation of three models.

Model	$Q^{2}$	$R^{2}$	SEE	F-Value	NOC	$R_{\mathrm{pred}\;}^{2}$	Fraction				
							S *	E *	H *	D *	A *
CoMFA	0.67	0.992	0.035	27.47	9	0.683	0.814	0.186	-	-	-
CoMSIA/SHE	0.69	0.974	0.059	15.52	7	0.758	0.069	0.135	0.797	-	-
CoMSIA/SEAH	0.66	0.975	0.057	12.30	7	0.767	0.067	0.138	0.779	-	0.016

* S: steric; E: electrostatic; H: hydrophobic; A: H-bond acceptor; D: H-bond donor.

**Table 4:** Recapitulation of some statistical parameters.

Statistical Parameters	CoMFA	CoMSIA/SEH	CoMSIA/SEAH
Q²	0.67	0.69	0.66
R² pred	0.683	0.758	0.767
K	0.923	0.922	0.923
K’	1.082	1.083	1.082

**Table 5:** Summary of the parameters of synthetic accessibility.

Numbers of Compounds	Characteristic						Violations			S.A
	MW	Nub-HA	Nub-HD	Nub-Rot	TPSA	LogP	Lipinski	Veber	Egan	
Criteria	<500	<10	<5	≤10	≤140	≤5	≤1	≤1	≤1	0 < S.A < 10
17	500.40	11	1	6	94.17	4.088	Yes	Yes	Yes	3.52
28	448.40	9	1	6	104.4	2.949	Yes	Yes	Yes	3.35

**Table 6:** The results of the ADMET test for two the most active molecules.

	Absorption			Distribution		Metabolism					Excretion	Toxicity	
	Water Solubility	Intestinal Absorption	Caco2 Permeability	VDss	CNS Permeability	Substrate		Inhibitor			Global Clearance	AMES Toxicity	Skin Sensitization
						CYP 450							
						2D6	3A4	1A2	2C19	2C9			
Unit	log mol/Liter	Percent %	log Pap 10^−6^ cm/s	Log Liter/kg	Log PS	Yes or No					Log mL/min/kg	Yes or No	Yes or No
17	−4.971	91.00	1.207	−0.374	−2.309	No	Yes	No	Yes	Yes	0.473	No	No
28	−4.119	85.348	1.378	−0.394	−3.079	No	Yes	No	No	No	0.293	No	No

VDss: volume of distribution; CNS: central nervous system; CYP 450: cytochrome p450.

## Data Availability

Not applicable.
